# Stress-Sensitive Protein Rac1 and Its Involvement in Neurodevelopmental Disorders

**DOI:** 10.1155/2020/8894372

**Published:** 2020-11-24

**Authors:** Xiaohui Wang, Dongbin Liu, Fangzhen Wei, Yue Li, Xuefeng Wang, Linjie Li, Guan Wang, Shuli Zhang, Lei Zhang

**Affiliations:** ^1^Department of General Surgery, Xuanwu Hospital, Capital Medical University, Beijing 100053, China; ^2^Peking University Hospital, Beijing 100870, China; ^3^Guangwai Community Health Service Center of Xicheng District, Beijing 100055, China; ^4^Department of Epidemiology, Emory University, Atlanta, GA, USA; ^5^School of Pharmaceutical Sciences, Tsinghua University, Beijing, China; ^6^State Key Laboratory of Brain and Cognitive Sciences, Institute of Biophysics, Chinese Academy of Sciences, Beijing 100101, China; ^7^Neurological Research Unit, Staidson (Beijing) Biopharmaceuticals Co., Ltd., Beijing 100176, China

## Abstract

Ras-related C3 botulinum toxin substrate 1 (Rac1) is a small GTPase that is well known for its sensitivity to the environmental stress of a cell or an organism. It senses the external signals which are transmitted from membrane-bound receptors and induces downstream signaling cascades to exert its physiological functions. Rac1 is an important regulator of a variety of cellular processes, such as cytoskeletal organization, generation of oxidative products, and gene expression. In particular, Rac1 has a significant influence on certain brain functions like neuronal migration, synaptic plasticity, and memory formation *via* regulation of actin dynamics in neurons. Abnormal Rac1 expression and activity have been observed in multiple neurological diseases. Here, we review recent findings to delineate the role of Rac1 signaling in neurodevelopmental disorders associated with abnormal spine morphology, synaptogenesis, and synaptic plasticity. Moreover, certain novel inhibitors of Rac1 and related pathways are discussed as potential avenues toward future treatment for these diseases.

## 1. Introduction

As a member of the Ras-homologous (Rho) small GTPase family, Rac1 is well known for its versatility in mediating the response of cells or organisms when facing external disturbances or environmental challenges, such as heat shock [1], oxidative stress [2], mechanical stress [3], genotoxic stress [4], hypoxic stress [5], or even higher-level mental stress from social confrontation and fear [6–9]. In the last decade, Rac1 has gained increased attention in the field of neuroscience with its roles in brain structure and function becoming more widely appreciated. It is commonly accepted that Rac1 and related signaling pathways are prominently involved in the maintenance and regulation of basic nervous system functions including neurite outgrowth, neuronal migration, synaptogenesis, synaptic plasticity, and learning memory [10–13]. Moreover, Rac1 is believed to contribute to the formation of addictive behavior [14]. However, not until recently have studies revealed that Rac1 may be relevant for certain inherited neurodevelopmental disorders, likely due to its essential role in the regulation of neuronal cell structure and development [15–19]. In this review, we aim to sketch a picture of the newly identified roles of Rac1 in these diseases and to shed light on the potential of specific inhibitors for Rac1 as novel therapeutics.

## 2. Basic Molecular Mechanism of Rac1 Signaling

**Figure 1:**
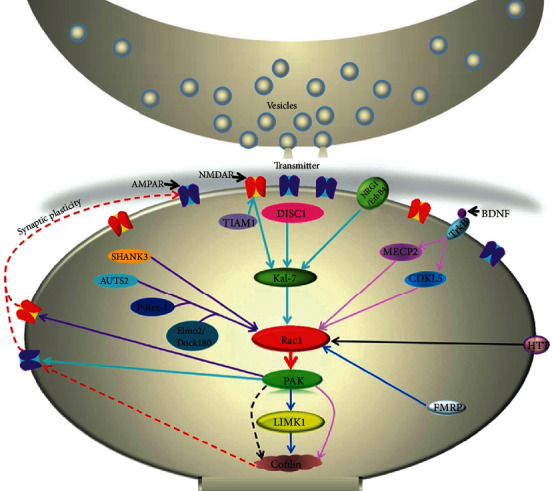
Regulation and interaction of Rac1-related signaling pathways at the postsynaptic terminal. Effectors of FXS and Huntington's disease, such as FMRP and HTT, can directly activate or inhibit Rac1 activity to modulate its downstream signaling cascades, mainly *via* the Rac1-PAK-cofilin pathway, which subsequently influences synaptic plasticity. In schizophrenia, NMDA receptors activate Kal-7 *via* TIAM1, while DISC1 and NRG1/ErbB4 interact with Kal-7 to activate or inhibit Rac1. In ASD, SHANK3 directly modulates Rac1 activity, while other effectors like AUTS2, P-Rex-1, and Elmo2/Dock180 form a complex to modulate Rac1 activity and then affect NMDA receptor activity through the PAK pathway. In Rett syndrome, BDNF activates TrkB receptors to modulate the activity of CDKL5 and MECP2 that further regulate the function of the Rac1-cofilin pathway. Abnormalities of these proteins in any pathways may affect neuroplasticity and cause neurodevelopmental disorders.

Rac1 belongs to the Rac subfamily of Rho small GTPases (~21 kDa), whose primary function is to transduce external signals to the inside of a cell. Rac proteins are among the frontline responders to external stress signals [20]. To date, three Rac proteins (Rac1–3) have been identified in vertebrates, which share a high degree of homology in amino acid sequences (88–92%) [21]. Rac1 participates in a wide spectrum of physiological processes, including actin cytoskeleton organization, cell adhesion and migration, gene expression, neurodevelopment, and synaptic plasticity [12, 22–24]. Rac1 was first identified in the human leukemia cell line HL-60 as a substrate of botulinum C3 ADP-ribosyltransferase [25, 26]. Similar to other small GTPases, Rac1 possesses a G core domain and an effector binding domain [27]. It is expressed in both the eukaryotic cytoplasm and the nucleus and cycles between the GTP-bound and GDP-bound states, marking the active and inactive forms of Rac1, respectively. To enter the active form, the bound GDP on Rac1 is replaced by GTP which is catalyzed by specific guanine nucleotide exchange factors (GEFs). Conversely, bound GTP is hydrolyzed to GDP by GTPase-activating proteins (GAPs) to produce the inactive form of Rac1 [28, 29]. Rac1 shares an identical amino acid sequence between murine, bovine, and human [30, 31]. The high degree of conservation with Rac1 protein structure and its downstream signaling cascades highlights its physiological relevance across different species. Rac1 exerts its functional impacts mainly *via* a downstream effector named p21-activated kinase (PAK). PAK directly phosphorylates and activates the LIM kinase (LIMK), which in turn phosphorylates and inactivates the actin-depolymerizing factor, cofilin, leading to actin depolymerization and cytoskeleton reorganization (Figure 1). In addition to the PAK-LIMK-cofilin pathway, Rac1 can also act directly through the WAVE1 and actin-related protein 2/3 (Arp2/3) complex to regulate actin nucleation and thus cellular structure, movement, and functions [32–36].

## 3. Rac1 and Neurodevelopmental Disorders

**Figure 2:**
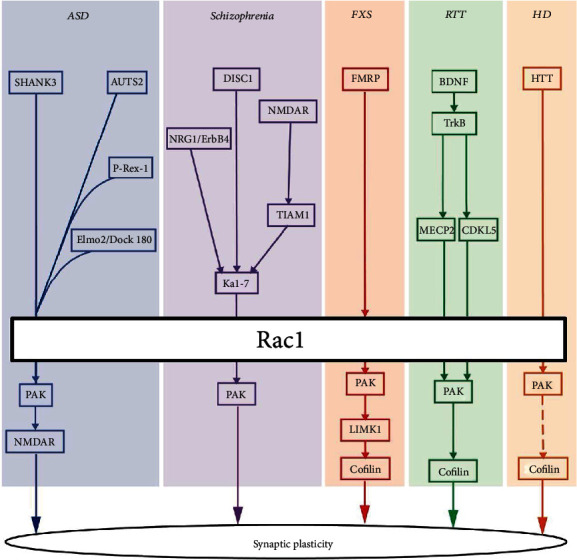
Putative schematic of the Rac1 signaling pathways involved in different neurodevelopmental disorders. The upstream effector, such as Kal-7, AUTS2, NMDAR, or FMRP, activates or inhibits Rac1 activity to modulate its downstream signaling cascades, primarily *via* the PAK-cofilin pathway, which orchestrates neuronal cell migration, spinogenesis, and synaptic plasticity. Abnormalities in this Rac1-related signaling complex are common features of neurodevelopmental disorders. The dashed lines indicate certain mechanisms that remain unclear and require further investigation.

Given the vital role that actin dynamics plays in multiple key physiological processes in the brain, it is no surprise that Rac1 influences a wide variety of nervous system functions, including synaptogenesis, neuronal migration, neurite outgrowth, synaptic transmission and plasticity, memory, and addictive behavior formation [14, 37–41]. Aberrant Rac1 expression or activity regulation or even small alterations to its downstream signaling may lead to severe neurodevelopmental disorders (Figure 2). Here, we briefly summarize recent findings on a few hereditary neurodevelopmental disorders that involve dysregulated Rac1 expression/activity, which have not been systemically covered previously in other reviews.

### 3.1. Autism Spectrum Disorders

Autism spectrum disorders (ASD) refer to a group of neurodevelopmental disorders characterized by impaired social interaction and communication along with restricted or repetitive behaviors [16, 42]. ASD can be diagnosed at any age; however, they are described as developmental disorders because they become apparent mostly in the first two years of life. A number of high-risk genes linked to the etiology of ASD have been identified and characterized, including the Autism susceptibility candidate 2 (AUTS2), SH3 and multiple ankyrin repeat domains 3 (SHANK3), Ubiquitin-protein ligase E3A (UBE3A), and Methyl-CpG-binding protein 2 (MECP2) [19, 43–49]. Some of these autism-risk genes were recently found to be linked to ASD *via* the Rac1-associated signaling network in the brain.

The *AUTS2* gene was first found to be disrupted by a *de novo* balanced translocation in two monozygotic twins with ASD [50]. It was later found to contribute to ASD by influencing synaptogenesis and neuron migration *via* Rac1. The AUTS2 protein activates Rac1 by interacting with different GEFs, such as P-Rex-1 and Elmo2/Dock180 complex, to promote the formation of lamellipodia in neurons [18, 43, 44]. Rac1 is also essential for AUTS2-mediated neuronal migration and neuritogenesis in the process of corticogenesis. At the early stages of cerebral cortex development, AUTS2 deficiency in mice results in retarded cortical neuronal migration that can be rescued by the overexpression of wild-type Rac1 [18, 44].

*SHANK3* is another highly studied risk gene for ASD. The link between *SHANK3* haploinsufficiency and ASD has been extensively studied in human and animal models [42, 51, 52]. *SHANK3*-deficient mice exhibit typical autistic cellular and behavioral phenotypes [52]. It was recently shown that the social deficits and diminished synaptic *N*-methyl-D-aspartate (NMDA) receptor function in *SHANK3*-deficient mice resulted from actin filament disorganization, caused by reduced Rac1/PAK activity and increased cofilin activity in the prefrontal cortex. To support this conclusion, it was found that both behavioral deficits and NMDA receptor malfunction were rescued by restoring Rac1/PAK activity in these mice [16, 45].

Impaired reversal learning caused by behavioral inflexibility is another hallmark symptom of ASD [53]. In a recent study with fruit flies (*Drosophila melanogaster*), Rac1 was a functional converging point for multiple autism risk genes, including Fragile X mental retardation 1 (*FMR1*), *UBE3A*, Neurexin-1 (*Nrx*-*1*), Neuroligin-4 (*Nlg4*), and Tuberous sclerosis complex 1 (*TSC1*). Mutations on these genes all caused similar autistic behavioral inflexibility related to Rac1-dependent memory impairment, which led to impaired reversal learning [47].

### 3.2. Schizophrenia

Schizophrenia is an inherited, severe psychiatric disorder that is thought to be associated with disturbances in neural network connectivity [54, 55]. An examination of postmortem brains from schizophrenia patients revealed the reduced density of dendritic spines and fewer glutamatergic synapses [56]. In the past decade, several schizophrenia risk genes have been reported, such as Disrupted-in-Schizophrenia 1 (*DISC1*), NMDA receptor subunit genes, Neuregulin 1/ErbB4 (*NRG1*/*ErbB4*), and Brain-derived neurotrophic factor (*BDNF*), which participate in the regulation of neuroplasticity and neural connectivity [15, 57–60]. Rac1 functions mostly as a downstream signaling hub molecule of these genes [11, 15, 57, 61].

Kalirin 7 (Kal-7) is a Rac1 GEF that was found to be transcriptionally downregulated in the prefrontal cortices of patients with schizophrenia [56]. Multiple lines of evidence support a critical role for Kal-7 in the modulation of spine morphology, driven mainly by the Rac1-dependent regulation of actin cytoskeleton [61–63]. Kal-7 interacts with DISC1 as a signalosome to control the duration and intensity of Rac1 activation in response to NMDA receptor activation. In rodent primary cortical neurons, DISC1 deficiency activates Rac1 and leads to rapid spine growth, while the overexpression of DISC1 suppresses Rac1 activity to reduce spine size [15]. Kal-7 also interacts with NR2B, an NMDA receptor subunit extensively involved in nervous system function and neurological diseases [10, 64–66]. It was noted that the NR2B-dependent NMDA receptor currents are diminished in neurons lacking Kal-7 [10, 61]. Additionally, Kal-7 is also involved in signaling cascades mediated by synaptic receptors like Ephrin B (EphB), Erb-B2 receptor tyrosine kinase 4 (ErbB4), and 5-Hydroxytryptamine (serotonin) receptor 2A (5HT2A), which regulate structural and functional plasticity of synapses [60, 62, 67, 68].

Like Kal-7, TIAM1 (TIAM Rac1-associated GEF 1) is a Rac1 GEF that colocalizes with the NR1 subunit of NMDA receptors. TIAM1 deficiency leads to reduced spine size, a result of TIAM1 binding to NMDA receptors and induction of local Rac1-dependent spine morphogenesis [69]. Moreover, other studies suggest that the inhibition of PAK prevents the progressive synaptic deterioration in rodent models of schizophrenia [57, 70, 71]. These studies collectively demonstrate a role for Rac1 and its regulators/effectors in the pathogenesis of schizophrenia, suggesting that Rac1 and related signaling pathways can be novel therapeutic targets.

### 3.3. Fragile X Syndrome

Fragile X syndrome (FXS) is a hereditary neurodevelopmental disability that results from an abnormal expansion of the CGG trinucleotide repeat in the *FMR1* gene on the X chromosome. The expansion leads to promoter hypermethylation and transcriptional silencing that prevents the expression of FMR1 protein (FMRP). FXS is characterized by abnormalities in dendritic spine structure, learning disabilities, and cognitive impairment. As an X-linked disorder, FXS occurs in males about two times more frequently compared to females [72–77].

Rac1 is found physically and functionally associated with FMRP. FMRP acts as a negative regulator of Rac1 synthesis [78]. Abnormally high Rac1 activity has been observed in the neocortices of FXS patients and animal models [79]. In the actin ring of murine fibroblasts, Rac1 colocalizes and interacts with FMRP and its partners, which serve as regulators of Rac1-dependent actin remodeling [80]. In developing *Drosophila* brains, Rac1 interacts with the homolog of FMRP to influence the cytoskeletal dynamics and neuronal outgrowth as well as synaptic morphology at neuromuscular junctions (NMJ). Loss-of-function mutations in FMRP increase the number of higher-order dendritic branches. Conversely, expression of normal FMRP dramatically decreases dendritic branching in *Drosophila* dendritic arborization (DA) neurons [81]. Comery et al. generated *FMR1* knockout (KO) mice and observed a larger proportion of long but thin dendritic spines in the occipital cortex with elevated Rac1, similar to what was observed in humans [79]. Another study demonstrated that Rac1 expression levels are unusually high in brain stems, hippocampi, and cortices of 3-month-old mice lacking *FMR1* [78].

Rac1-related signaling pathways have also been extensively investigated in FXS animal models. When Rac1 is absent in the synapses of certain brain regions, PAK activity is also downregulated. Partial inhibition of PAK by introducing dominant-negative PAK in mice results in a shift in the overall spine distribution toward shorter spines with a lower proportion of longer spines relative to wild-type neurons [82]. Opposite phenotypes have been observed in *FMR1* KO mice [78]. In a separate study, the application of a small-molecule PAK inhibitor FRAX486 rescued most of the FXS phenotypes in *FMR1* KO mice [83]. The activity of cofilin was also found to be suppressed in the somatosensory cortex of *FMR1* KO mice, due to the hyperactivity of Rac1 [84]. These studies revealed a previously unappreciated role for impaired Rac1-PAK1-cofilin-LIMK1 signaling in abnormal spine morphology and density associated with FXS and pointed to Rac1 as a promising target for these specific abnormalities.

### 3.4. Rett Syndrome

Rett syndrome (RTT) is a severe progressive developmental intellectual disability that affects almost exclusively girls [85, 86]. Autopsy studies of six girls that died between the ages of 2.9 and 35 revealed excessive amounts of immature dendrites in the motor and frontal cortices [87]. Two high-risk genes linked to RTT have been identified: *MECP2* and the X-linked cyclin-dependent kinase-like 5 (*CDKL5*). It was reported that mutations on *MECP2* account for ~20% of classical RTT along with 60~80% of RTT variants [88, 89], while mutations on *CDKL5* were identified in patients with the Hanefeld variant of RTT [90, 91].

In patients with *MECP2* mutations, significant decreases in spine density were observed in hippocampal CA1 pyramidal neurons. Similarly, reductions of spine density were observed in the motor cortices and hippocampi of mice lacking *MECP2* [92–94], which was found to be at least partially attributed to the deregulation of BDNF, a protein well known for its vital role in spine growth and synaptogenesis by binding and activating the tropomyosin-receptor kinase B (TrkB) receptor and its downstream signaling pathways [95–99]. Although more evidence is needed, Rac1 has been proposed to act as a downstream signaling effector of BDNF [100, 101]. Moreover, in the brains of RTT model mice, activation of certain Rac1 downstream proteins such as PAK and cofilin directly activates mTOR in *MECP2* mutant neurons, which is responsible for the translational control of altered proteins in RTT [102].

Patients with *CDKL5* mutations exhibit prominent intellectual disabilities. Knocking down *CDKL5* in the rat brain results in delayed neuronal migration and severely impairs dendritic arborization. Overexpression of Rac1 rescues the dendritic growth inhibited by CDKL5 knockdown. Moreover, CDKL5 is required for the BDNF-induced activation of Rac1 [103, 104].

Taken together, Rac1 mediates the dendritic development through BDNF regulation, which may be a common mechanism in cases of RTT involving *MECP2* or *CDKL5* mutations. Rac1 is also responsible for the posttranslational control of altered proteins in cases of RTT involving *MECP2* mutations. The modulation of Rac1-dependent spine development is potentially beneficial as a treatment for RTT.

### 3.5. Huntington's Disease

Huntington's disease (HD) is a hereditary and progressive nervous system disorder that is caused by a CAG trinucleotide repeat expansion in the first exon of the *HTT* gene, which encodes for the huntingtin protein (HTT) [105]. HD patients manifest with progressive motor, cognitive, and emotional impairments, coupled with abnormal spine morphogenesis in the cortex and striatum [106–108].

Recent studies suggest that Rac1 may contribute to the pathogenesis and symptoms of HD. In a large-scale screening study with the yeast two-hybrid system, over 2000 HTT-interacting proteins were analyzed. A few Rho GTPase signaling components, including Rac1 and PAK2, were identified as modifiers of mutant HTT toxicity, which implicates Rac1 and related signaling cascades in the onset of HD [109]. Consistent with this screen, Rac1 activity was found to be drastically enhanced in both primary human fibroblasts lacking HTT and the striatum of 1.5-month-old HD Q140/Q140 knock-in mice [110, 111]. Moreover, consistent with the conventional role of Rac1 in oxidative stress, multiple recent studies suggest that Rac1 modulates the generation of reactive oxygen species (ROS) in HD models [112, 113]. Nevertheless, even though studies suggest that Rac1 is involved in actin-dependent morphological changes of neurons during the pathogenesis of HD [110], whether Rac1 contributes to the crucial early development of HD remains unclear and requires further investigation.

## 4. Screening Novel Rac1 Inhibitors for the Treatment of Neurodevelopmental Disorders

Due to the multifaceted roles that Rac1 plays in brain function and neurodevelopmental disease etiology, enormous efforts have been made to screen for effective Rac1 inhibitors with the hope to develop novel medications for relevant neurodevelopmental diseases (Table 1). A good number of small-molecule compounds targeting Rac1 and related signaling pathways have been developed. NSC23766 and its derivatives have been extensively investigated in multiple disease models, both *in vitro* and *in vivo*. NSC23766 inhibits Rac1 activity by disrupting its physical binding with its interacting proteins, such as GEFs, TIAM1, and triple functional domain protein (TRIO), without affecting RhoA or Cdc42 activity. Preclinically, NSC23766 has demonstrated positive effects in several disease models, including in models of cancers, cognitive disorders, brain injuries, and neurodegenerative and kidney diseases [112–118]. Based on its structure and characteristics, further optimization of NSC23766 has been performed. AZA1 is a compound structurally based on NSC23766 that has greater inhibitory potency on Rho GTPase activity, which was shown to inhibit both Rac1 and Cdc42 activity in prostate cancer cells and improve the survival rate of mice bearing human prostate cancer xenografts [119]. EHop-016 is another potent Rac inhibitor derived from NSC23766 that targets the association between Rac1 and the Rac GEF Vav2. EHop-016 is highly effective at inhibiting Rac1 activity and suppressing Rac-directed lamellipodia formation in MDA-MB-435 metastatic cancer cells and MDA-MB-231 metastatic breast cancer cells [120].

Even though much work has been done to optimize NSC23766-based molecules, a big gap still remains with further development into clinical testing, possibly due to their low binding capacity to the target proteins.

Unraveling the crystal structures of Rac1 and its interacting proteins opened another door to identify inhibitory compounds [121]. ITX3 was identified as a selective inhibitor of TRIO and N-terminal TRIO-dependent cell structures *in vitro* based on the crystal structures and characteristics of the target proteins. However, the efficacy of ITX3 in animal models appears to be not ideal [122]. A thorough screening of the ZINC database containing more than 200,000 compounds led to the discovery of a number of novel Rac1 inhibitors. 1A-116 shows a robust antimetastatic effect by blocking Rac GEF P-Rex-1 (PIP3-dependent Rac exchanger 1) binding to suppress Rac1 activation [123]. In addition, ETH 1864 was identified to be a potent suppressor of Rac1a and its GEF, TIAM1, as well as the other Rac1 isoforms, Rac1b, Rac2, and Rac3. ETH 1864 has been demonstrated to decrease the NMDAR current density in rat cortical neurons and reduce spine density in the early stages of hippocampal neuronal development [124]. Consistent with this, LTP and LTD were both abolished by the treatment of ETH 1864 and NSC23766, respectively, on mouse hippocampus slices [11]. The downstream effectors of Rac1, such as PAK, have also been targets for novel inhibitor screening. Through a traditional, two-part structure-activity relationship approach, a series of PAK inhibitors were found in a library of 12,000 compounds using a FRET-based assay. Among them, FRAX486 was identified to show a potent inhibitory effect to PAK with good PK properties and brain penetration. The small molecule was demonstrated to reverse the spine abnormalities seen in animal models of FXS and schizophrenia. Behavioral phenotypes, such as hyperactivity and repetitive movements, were also rescued after treatment with FRAX486 [71, 83].

So far, although no small-molecule inhibitors have moved into clinical trials, recent alternative approaches to targeting Rac1 offer more optimism for this approach. A polypeptide that inhibits Rac1 and a few other RhoA GTPases is in preclinical testing with positive effects shown on treating neurodegenerative diseases and cancer [125]. An antisense RNAi oligonucleotide drug for Kaposi's sarcoma developed by researchers from the University of Miami has also been reported [126]. Although no drugs have been tested in humans at this moment, the application of these Rac1 inhibitors in neurodevelopmental disease models may provide new leads for diseases featuring synaptic abnormalities.

## 5. Conclusions

The dysregulation of Rac1 has been indicated in the processes of neuronal morphogenesis, migration, and synaptic plasticity in neurodevelopmental disorders such as schizophrenia, ASD, and FXS, as highlighted in the review. Given the lack of effective medications for these diseases, Rac1 presents an opportunity for therapeutic intervention by targeting the abnormalities in synaptic morphology and plasticity. Further exploration of the expression and modulation of specific Rac1 regulators as well as Rac1 itself under physiological and pathological conditions will be beneficial to our understanding of the underlying mechanisms of these diseases as well as the development of novel therapeutic approaches.

## Figures and Tables

**Table 1:** List of small-molecule compounds that inhibit Rac1 activity.

Compound name	Formula	Molecular weight	Target Rac1 signaling	Target other Rho GTPases	Reference
NSC23766	C_24_H_35_N_73_HCl	530.96	Inhibit TIAM1 and TRIO	None	[114]
ITX3	C_22_H_17_N_3_OS	371.45	TRIO	RhoG and Rac1	[122]
EHop-016	C_25_H_30_N_60_	430.55	Inhibit Vav2	Rac3, Cdc42	[120]
AZA1	C_22_H_20_N_6_	368.43	Rac1/PAK1	Cdc42	[119]
1A-116	C_16_H_16_F_3_N_3_	307.31	Rac1/P-Rex-1	None	[123, 127]
ETH 1864	C_25_H_27_F_3_N_204_S_2_HCI	581.47	Rac1/TIAM1	Rac1b, Rac2, Rac3	[128]
FRAX486	C_25_H_23_C_l2_FN_6_O	513.39	PAK1-3	None	[71, 83]

## Data Availability

No data were used to support this study.
